# High burden of hypertension across the age groups among residents of Gondar city in Ethiopia: a population based cross sectional study

**DOI:** 10.1186/s12889-017-4646-4

**Published:** 2017-08-09

**Authors:** Abayneh Girma Demisse, Ermias Shenkutie Greffie, Solomon Mekonnen Abebe, Abera Balcha Bulti, Shitaye Alemu, Bewketu Abebe, Nebiyu Mesfin

**Affiliations:** 1 School of Medicine, College of Medicine and Health Sciences, University of Gondar, Gondar, Ethiopia; 2 Institute of Public Health, College of Medicine and Health Sciences, University of Gondar, Gondar, Ethiopia

## Abstract

**Background:**

According to a report on the worldwide trends in blood pressure from 1975 to 2015, mean blood pressure is increasing in low and middle income countries while it is either decreasing or stabilizing in high income countries. Few studies have been published on the prevalence of hypertension in Ethiopia demonstrating an increased trend; however, these studies had small sample size and were limited to participants older than 35 years; which left the burden among adolescents and young adults unaddressed. The aim of this study was to assess prevalence of hypertension (HTN) and associated factors in Gondar city.

**Method:**

A population based cross-sectional study was conducted among 3227 individuals in Gondar city. A multistage cluster random sampling was used. The Kish method from World Health Organization (WHO) STEPS instrument of random sampling method was used for selecting one individual older than or equal to 18 years from each household. WHO and International Diabetic Association (IDA) criterion was used to classify HTN.

**Result:**

The overall prevalence of HTN was 27. 4% [95% CI: (25. 8–28.9)]. The prevalence for participants in the age group of ≥35 years was 36. 1%. It consistently increased from 9.5% in the age group of 18–25 years to 46.3% in the age group of ≥65 years (*P*-value < 0. 001). Only 47% of the participants had ever had any kind of blood pressure measurement. Being elderly (AOR = 5. 56; 95% CI: 3. 71–8. 35), obese (AOR =2. 62; 95%CI: 1. 70–4. 03), widowed (AOR = 1. 87; 95%CI: 1. 27–2. 75), separated (AOR = 1. 87; 95%CI: 1. 27–2. 75), daily alcohol user (AOR = 1. 51; 95%CI: 1. 02–2. 23), male gender (AOR = 1. 42; 95%CI: 1. 18–1. 72) and born in urban area (AOR = 1. 31; 95%CI: 1. 10–1. 56) were found to be independently associated with HTN.

**Conclusion:**

There is a high prevalence of hypertension in Gondar city and is showing increasing trend compared to previous reports. Interventions to raise awareness and to improve both capacity and accessibility of facilities for screening hypertension are highly recommended.

## Background

The global burden of disease, injuries and risk factors study in 2015 reported that high blood pressure was the leading risk factor contributing for about 211. 8 million of the global disability adjusted life years(DALYs) [1]. It was also the most common risk factor for death due to cardiovascular disorders, chronic kidney disease and diabetes causing more than 40% of the deaths due to these disorders globally [2]. According to a report on the worldwide trends in blood pressure from 1975 to 2015, mean blood pressure is increasing in low and middle income countries while it is either decreasing or stabilizing in high income countries [3]. In 1975 the highest mean blood pressure was in high income western and Asia Pacific countries. After 40 years, mean blood pressure has significantly decreased in those high income countries. In 2015, the high income countries had the lowest recorded blood pressure while Sub-Saharan Africa (SSA) had the highest in the world [3].

Sub-Saharan Africa (SSA) is currently undergoing an epidemiologic transition from one which was dominated by infectious disease to one dominated by non-communicable disease including hypertension. Hypertension was among the major risk factors for cardiovascular disease in Africa and was found to be associated with high mortality [4]. Although it is difficult to compare the studies due to the heterogeneity of the population studied, prevalence rates reaching up to 38% have been reported in different SSA countries [5–7].

In Ethiopia, non-communicable diseases including hypertension were not given as much attention as communicable diseases in the past. This was in part due to the reported low prevalence of hypertension in older studies ranging between 0.4 and 11% [8–10]. However, the relatively recent studies have shown the prevalence to be progressively growing ranging from 16.9% to as high as 31.5% especially in the urban population [11–13]. A study conducted in Addis Ababa to evaluate the causes of death in hospitals during 2002–2010 found out that cardiovascular disorders accounted for 11% of the total deaths which was equivalent with deaths from HIV/AIDS [14].

There were few previous studies on hypertension prevalence in the study area. One study conducted in 2012 in Gondar city reported a prevalence of 28% [13]. Another study which aimed at assessing the urban rural gradient showed prevalence of 30.7 and 25.3% in urban and rural are as respectively [15]. However, unlike the current larger scale and more inclusive study, those previous studies had a relatively small sample size and included only adults above the age of 35 years.

This study was conducted as part of a bigger project aimed to assess prevalence of cardiovascular risk factors in Gondar town. The study was one of its kinds in the area as it included huge number of participants and detailed inquiry and measurement of not only blood pressure but also blood sugar levels and lipid profiles was performed according to the WHO STEP wise approach.

## Methods

### Study area and population

This study was conducted in Gondar town, in the North-west part of Ethiopia located 750 Km from the capital Addis Ababa. It is one of the largest cities in the country with a population of 358, 257. A community based cross sectional study design was implemented. All individuals of age greater than or equal to 18 years who reside in Gondar city for at least six months were included. The sample size for the study was determined by assuming 50% prevalence with a 95% confidence interval and 5% margin of error. We also consider a 5% non-response rate. After having the minimum sample size stratification of the sample for age and sex was done (four age categories multiplied by two (for male and female), accordingly, the calculated sample size was 3227 individuals.

A multistage cluster random sampling method was used. At first, four administrative kebeles (the smallest administrative units in Ethiopia which consists of at least 500 families) were randomly selected from the 12 administrative kebeles using simple random sampling after obtaining the list from the Gondar city administration. Households were selected within each administrative kebeles using the systematic random sampling technique. Finally, eligible adult from each group until the required sample was selected from each household using simple random sampling. In cases where there were more than one eligible individual in the selected household, a lottery method was used to pick one (The Kish method from WHO step instrument of random sampling method was used for selecting one individual from each household).

All the individuals were given an identification number and a household number by the data supervisor.

### Data collection procedure

Data were collected by interviewing eligible subjects using a structured questionnaire. House-to-house data collection was performed by trained nurses. The field study team was composed of enumerators, laboratory technicians, Health extension nurses, and supervisors. All were trained by the principal investigators for two days on the study procedures. To ensure the quality of the interview the supervisors checks 5 to 10% of daily collected the questioners.

### Measurements

WHO’s STEP-wise approach was used only in Gondar which is one of the largest towns in the country. We implemented the WHO and International Diabetic Association (IDA)criterion to classify hypertension with systolic blood pressure (SBP) of ≥140 mmHg and /or a diastolic blood pressure (DBP) ≥ 90 mmHg or known hypertensive patients on treatment. Isolated systolic hypertension (ISH) was defined as having a systolic blood pressure ≥ 140 mmHg and diastolic blood pressure < 90 mmHg and Isolated diastolic hypertension (IDH) was defined as having a systolic blood pressure < 140 mmHg and diastolic blood pressure ≥ 90 mmHg). Blood pressure (BP) was measured using a digital measuring device with participants sitting after resting for at least five minutes. Three BP measurements were taken with at least three-minute intervals between the consecutive measurements. The mean systolic and diastolic BP from the second and third measurements was considered for analyses [16]. Finally, biochemical tests (fasting blood glucose (FBG), triglyceride, LDL, HDL and total cholesterol test) were carried out [17]. Blood samples were collected from each participant by a trained laboratory technician following aseptic techniques. The blood samples were taken to the hospital laboratory for chemistry analyses. Biochemical tests were carried out using 902 Automatic Analyzer with following a minimum of 8 h fasting period, early in the morning before participants took their breakfast. [18, 19]. We used a ‘yes’ and ‘no’ question to extract history of smoking and alcohol consumption.

The WHO’s STEP-wise instrument approach was employed in the questions that assessed hypertension risk factors [16]. Anthropometric measurements were taken using standardized techniques and calibrated equipment. Subjects were weighed to the nearest 0.1 kg in light indoor clothing and bare feet or with stockings. Height was measured using a stadiometer; participants stood in erect posture without shoes, and the results were recorded to the nearest 0. 5 cm. Measures were taken two times, and the average was considered in the analysis. BMI was used to define underweight (BMI < 18. 4), normal (18. 5 ≤ BMI < 24. 0), overweight (25. 0 ≤ BMI < 29. 0), and obese (BMI ≥ 30) adults [17].

### Data analysis

Data entry procedures were done using the EPI Info version 3.5.3 statistical software. A stratified analysis was also performed to look at age, residence and sex specific proportion. The prevalence estimation was made along with a 95% confidence interval (CI). The results were considered statistically significant at *P* ≤ 0. 05. Logistic regression was applied to identify the associated factors with hypertension. To fit the multivariate analysis, independent variables were selected based on the conceptual framework and prior evidences in the literature and their effect in the current analysis using bivariate analyses; a cut of point *p*-value <0.20 was included. The independent variables, like socio-demographic factors and health related life-style characteristics of the study population were computed in the multivariable logistic regression analysis. Statistical analysis was performed using STATA version 14 software.

### Ethical approval

The protocol was approved by the Institutional Review Board (IRB) of the University of Gondar. In addition, a written permission was obtained from the respective local administration and hospital director. Participants were recruited voluntarily after obtaining full information about the research and signed a written consent agreement. They were informed of their rights to withdraw from the study at any stage. For the sake of privacy and confidentiality no personal identifiers such as names were collected.

## Results

A total of 3227 subjects were asked to participate, 168 of them refused, giving a response rate of 94. 8%. The mean age of the population was 41.1 ± 18.5 years and females accounted for 54.1% of the group. More than half of the patients (56. 9%) were born and raised in rural areas. About 90.9% of the participants were Orthodox Christians. About 53% of the participants did not have any kind of formal education. The socio-demographic characteristics of the study population are depicted in Table 1.

**Table 1:** Socio-demographic profiles of Gondar city residents who were ≥18 years old, Northwest Ethiopia

Variable	Male n (%)	Female n (%)	Total n (%)
Age in Years			
18–24	264 (47. 2)	295 (52, 8)	559 (18. 3)
25 to 34	305 (46. 8)	347 (53. 2)	652 (21. 3)
35 to 44	253 (43. 4)	330 (56. 6)	583 (19. 1)
45 to 54	236 (46. 3)	274 (53. 7)	510 (16. 7)
55 to 64	163 (42. 3)	223 (57. 8)	386 (12. 6)
≥ 65	185 (50. 1)	184 (49. 9)	369 (12. 1)
Location Birth			
Urban	633 (48. 1)	687 (51. 9)	1317 (43. 1)
Rural	773 (44. 4)	969 (55. 5)	1742 (56. 9)
Education status			
Unable to read and write	175 (44. 9)	214 (55, 01)	389 (12. 72)
Can read and write	590 (47. 7)	647 (52. 3)	1237 (40. 4)
Primary school	146 (53. 7)	126 (46. 3)	272 (8. 9)
Secondary school	117 (70. 5)	49 (29. 5)	166 (5. 43)
Diploma	13 (61. 9)	8 (38. 1)	21 (0. 69)
Degree and above	347 (38. 9)	545 (61. 1)	892 (29. 16)
Marital Status			
Single	483 (55. 8)	383 (44. 2)	866 (28. 3)
Married	832 (48. 2)	896 (51. 8)	1728 (56. 5)
Separated	21 (17. 1)	102 (82. 9)	123 (4. 02)
Divorced	29 (238)	93 (76. 2)	122 (3. 99)
Widowed	41 (18. 6)	179 (81. 4)	220 (7. 19)
Religion			
Orthodox	1230 (45. 5)	1473 (54. 5)	2, 703 (90. 9)
Muslim	129 (48. 9)	135 (51. 1)	264 (8. 9)
Protestant	4 (66. 7)	2 (33. 3)	6 (0. 20)
Work status over the last 12 month			
Government employee	201 (56. 8)	153 (43. 2)	354 (11. 57)
Private employee	100 (60. 2)	66 (39. 8)	166 (5. 43)
Personal Job	715 (58. 4)	509 (41. 6)	1224 (40. 01)
Non paid Job	5 (11. 1)	40 (88. 9)	45 (1. 47)
Student	162 (52. 3)	148 (47. 7)	310 (10. 13)
Home worker	3 (5. 3)	54 (74. 7)	57 (1. 86)
Retired	53 (70. 7)	22 (29. 3)	75 (2. 45)
Able to work	132 (20. 0)	528 (80. 0)	660 (21. 58)
Unable to work	35 (20. 8)	133 (79. 2)	168 (5. 49)
Did you ever had your BP measured for any reason			
Yes	609 (43. 3)	834 (50. 5)	1443 (47. 2)
No	797 (56. 7)	819 (49. 5)	1616 (52. 8)

Current smokers accounted for only 1. 84% of the population while 86. 7% of the participants either do not drink alcohol or drink less than 3 times per month. Fasting was a common religious practice in 22.3% of the participants.

The majority of participants had a normal BMI (61. 9%) while 13. 3% were underweight. Prevalence of obesity and overweight were 5.7 and 19.1% respectively. (Table 2).

**Table 2:** Behavioral and clinical profiles of Gondar city residents who were ≥18 years old, Northwest Ethiopia

Variable	N	%	Hypertension n (%)	P- value
Birth place				
Rural	1742	56. 9	452 (25. 9)	0. 001
Urban	1317	43. 1	385 (29. 2)	
Current Smoking				
Yes	56	1. 84	19 (33. 9)	0. 26
No	2984	98. 16	812 (27. 2)	
Alcohol Consumption in the past 12 months				
Daily	140	4. 58	53 (37. 9)	0. 011
5–6 days/week	64	2. 09	9 (14. 1)	
1–4 days/week	202	6. 60	59 (29. 2)	
1–3 days/month	642	24. 3	208 (28. 03)	
< once a month	466	15. 2	120 (25. 8)	
none	1445	47. 2	338 (26. 9)	
Work involving moderate activity				
Yes	777	25. 44	213 (27. 4)	0. 67
No	2, 277	74. 56	624 (27. 4)	
Religious Fasting practice				
Yes	2376	77. 7	617 (25. 9)	0. 001
No	683	22. 3	220 (32. 2)	
Body mass index kg/m^2^				
Underweight	406	13. 3	68 (16. 8)	<0. 001
Normal	1896	61. 9	485 (25. 6)	
Overweight	583	19. 1	213 (36. 5)	
Obese	174	5. 69	71 (40. 8)	
Mean and Standard Deviation (Mean ± SD)				
Total Cholesterol (mg/dl)	165. 8 (± 48. 4)			
Fasting blood glucose (mg/dl)	80. 7 (± 28. 2)

**Table 3:** Multivariate analysis for factors associated with hypertension among Gondar city residents who were ≥18 years old, Northwest Ethiopia

Variable	HTN n (%)	Adjusted OR [95%CI]
Sex		
Female	429 (25. 9)	1. 00
male	408 (29. 0)	1. 42 (1. 18, 1. 72)
Age in Years		
18–24	53 (9. 5)	1. 00
25–34	117 (17. 9)	1. 83 (1. 27, 2. 64)
35–44	153 (26. 2)	2. 56 [1. 75, 3. 73)
45–54	186 (36. 5)	3. 92 (2. 68, 5. 75)
55–64	157 (40. 7)	4. 68 (3. 16, 6. 94)
≥ 65	171 (46. 3)	5. 56 (3. 71, 8. 35)
Birth place		
Rural	452 (25. 9)	1. 00
Urban	385 (29. 2)	1. 31 (1. 10, 1. 56)
Marital status		
Single	142 (16. 4)	1. 00
Married	496 (28. 7)	1. 03 (0. 80, 1. 33)
Separated	51 (41. 5)	1. 87 (1. 27, 2. 75)
Divorced	41 (33. 6)	1. 09 (0. 69, 1. 75)
Widowed	107 (48. 6)	1. 87 (1. 27, 2. 75)
Alcoholic use in the last 12 months		
Daily	53 (37. 9)	1. 51 (1. 02, 2. 23)
5–6 days / week	9 (14. 1)	0. 47 (0. 22, 0. 98)
1–4 days /week	59 (29. 2)	1. 12 (0. 79, 1. 59)
1–3 days /week	208 (28. 03)	1. 01 (0. 81, 1. 25)
< once / month	120 (25. 8)	1. 03 (0. 79, 1. 34)
No alcohol in 12 months	338 (26. 9)	1. 00
Religious fasting practice		
Yes	617 (25. 9)	1. 00
No	220 (32. 2)	1. 16 (0. 95, 1. 42)
BMIkg/m2		
Under weight	68 (16. 7)	1. 00
Normal weight	485 (25. 6)	1. 51 (1. 12, 2. 03)
Over-weight	213 (36. 5)	2. 29 (1. 64, 3. 19)
Obese	71 (40. 8)	2. 62 (1. 70, 4. 03)
Total Cholesterol (mg/dl)	--	1. 00 (1. 00, 1. 01)
Fasting blood sugar (mg/dl)	--	1. 01 (1. 00, 1. 01)

### Blood pressure measurement

**Fig. 1:**
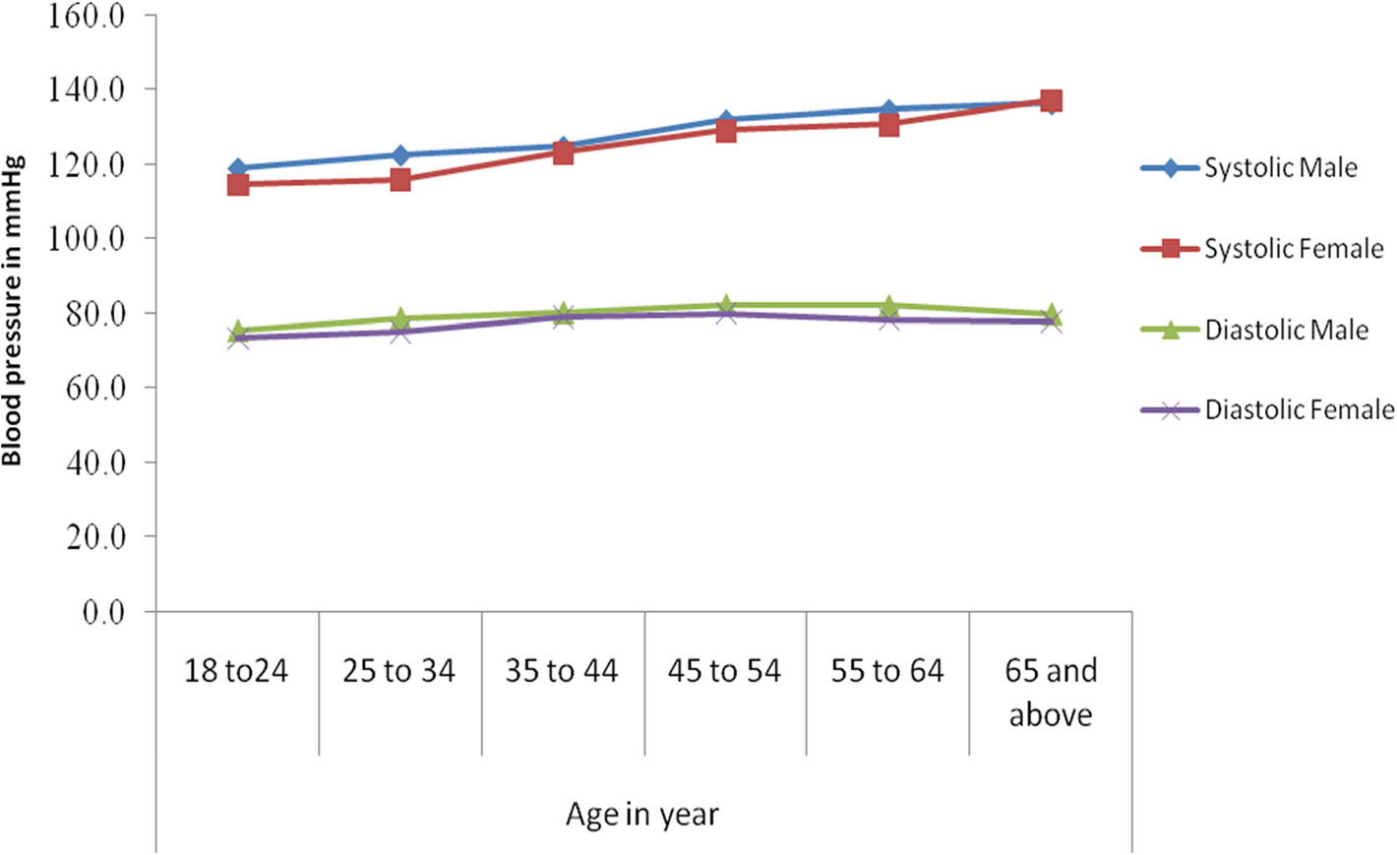
Mean Systolic and diastolic blood pressure by age and sex among residents of Gondar city

The mean (±SD) systolic and diastolic blood pressures were 125.3 (±19. 8) mmHg and 78.5 (±11. 4) mmHg, respectively. SBP was found to increase progressively with age in both sexes; whereas, the DBP was found to progressively increase only up to the age of 45–54 years of age and stabilizes thereafter in both sexes (Fig. 1). The sex specific mean (±SD) of SBP and DBP were 127 (±18. 4) and 79.9 (±11. 6) in males and 123.7 (±20. 7) and 77.3 (±11) in females, correspondingly.

### Prevalence of hypertension

**Fig. 2:**
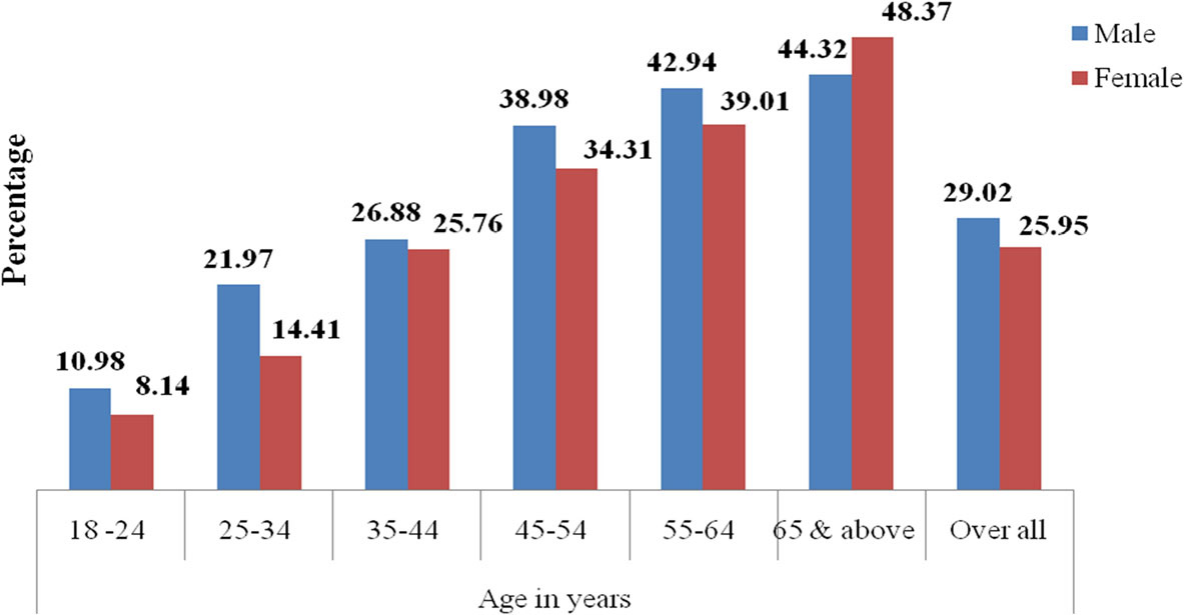
Prevalence of hypertension by sex and age among residents of Gondar city

The overall prevalence of HTN was 27. 4% [95% CI: 25. 8%-28. 9%]. The prevalence consistently increased from 9.5% in the 18–25 year group to 46.3% in those above 65 years (*p*-value <0. 001). The prevalence in participants above 35 years was 36. 1%. Overall, hypertension was more common in males (29%) than females (25. 4%) (*p*-value = 0. 05). This difference was maintained up to the age of 65 years. However, in participants above 65 years hypertension was more common in females (48. 4%) than males (44. 3%) as illustrated in Fig. 2.

**Fig. 3:**
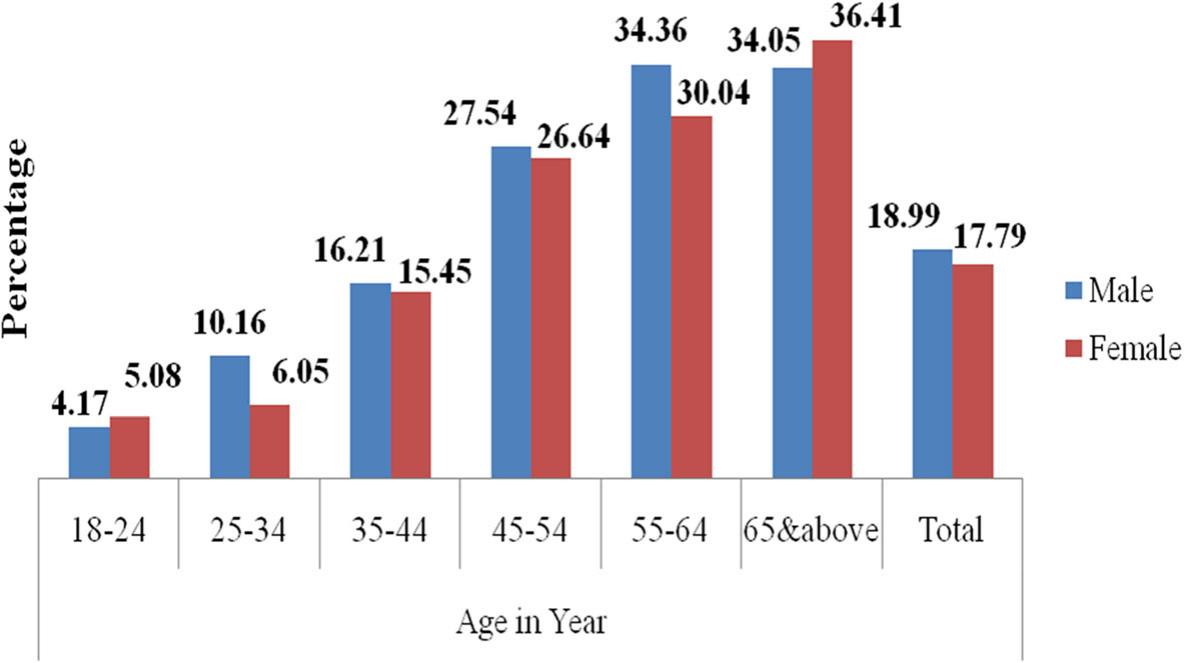
Prevalence of isolated systolic hypertension by age and sex among residents of Gondar city

The overall prevalence of ISH was 9. 4% [95%CI: 8. 4%-10. 5%]. It progressively increased with increasing age, from 2.7% in participants of age 18––24 years to 23.4% in those above 65 years. Figure 3 shows that the sex specific prevalence was consistently higher in males than females in participants of age 25–65 years. But, above the age of 65 years more females (26. 1%) than males (20. 5%) had ISH.

**Fig. 4:**
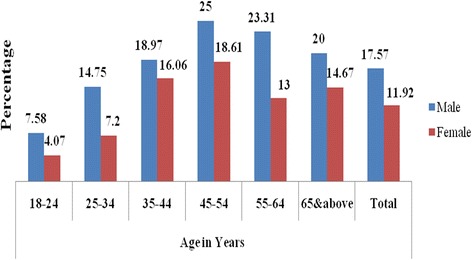
Prevalence of isolated diastolic hypertension by age and sex among residents of Gondar city

The overall prevalence of IDH was 5. 5% [95%CI: 4. 7%-6. 4%]. Figure 4 shows that it peaked in the 25–34 years group with a prevalence of 7%. It was higher in males compared to females across all age groups.

### Screening, treatment and control of hypertension

Based on the participants recall only 1443 (47. 2%) of the study participants, 43. 3% among males and 50. 5% among females, had history of ever getting a blood pressure measurement in their life time regardless of the purpose of measurement. Only 229 (7. 1%) of the participants were already diagnosed with hypertension and were taking anti-hypertensive treatment. Of the patients who were on antihypertensive treatment 128 (55. 9%) had controlled hypertension at the time of the study (BP < 140/90 mmHg). Out of the 876 hypertensive cases in the study, 72.6% were not aware of their hypertension status until they were diagnosed during this survey.

### Factors associated with hypertension

The factors significantly associated with hypertension among the non-modifiable risk factors were age, sex, place of birth and marital status. The male gender compared to females was independently associated with hypertension (AOR = 1. 42; 95% CI: 1. 18, 1. 72). Increasing age was strongly associated with hypertension. The odds of hypertension was more than 5 times higher among participants who were older than 65 years compared to those in the age group of 18–24 years (AOR = 5. 56; 95% CI: 3. 71, 8. 35). Individuals who were born and raised in the urban area also exhibited a 33% higher odds of having hypertension compared to those born and raised in rural areas, (AOR = 1. 33; 95% CI, 1. 10, 1. 56). Being widowed (AOR = 1. 87; 95%CI: 1. 27, 2. 75) and being separated (AOR = 1. 87; 95% CI: 1. 27, 2. 75) were found to be independently associated with hypertension compared to having a single marital status.

Among the conventional modifiable risk factors, obesity (AOR = 2. 62; 95% CI: 1. 70, 4. 03) and overweight (AOR = 2. 29, 95% CI: 1. 64, 3. 19) were found to be independently associated with hypertension compared to underweight. Compared to being underweight, even having a normal BMI was associated with higher occurrence hypertension (AOR = 1. 51; 95%CI: 1. 12, 2. 03). Compared to individuals who have never used alcohol in the preceding twelve months, participants with a history of daily alcohol use in the preceding year had independent association with hypertension (AOR = 1. 51; 95%CI: 1. 02, 2. 23) (Table 3).

## Discussion

This study was part of a population survey to assess cardiovascular risk factors in residents of Gondar city using WHO STEPS instrument in a big sample of 3227 participants of age ≥ 18 years stratified by age and sex. Previous community based studies on hypertension conducted in the area utilized a smaller sample size and age groups limited to a certain study groups mostly older than 35 years.

Unlike the age-specific prevalence of hypertension which was found to be as high as 46% in the age group of ≥65 years and as low as 9. 5% in the age group ≤25 years, the overall prevalence of hypertension in this study was found to be 27. 4% which is slightly lower than the findings in the previous studies 28.3% and 30.7% conducted in the same area [13, 15]. The prevalence in this study was also lower than the finding from a community based study done ten years ago in Addis Ababa, Ethiopia which showed a prevalence of 31.5% in males and 29.3% among women [11]. However, those previous studies didn’t include the younger age groups and the participants were older than 35 years of age whereas this study included all individuals ≥18 years. Therefore, the total exclusion of young adults in the previous studies could be responsible for the slightly high overall prevalence of hypertension. When the prevalence of HTN was calculated for age groups above 35 years in this study, the prevalence raised to 36.1% which is in fact higher than the previous studies.

A study done five years ago among Bank employees and teachers in Addis Ababa with no adolescent and few young adult participants found prevalence of HTN to be 19. 1% [20]. A similar study conducted three years ago in another town in the country with participants older than 30 years showed a prevalence of 22. 5% in males and 19% in females among urban population [12]. The reported prevalence in rural areas of the country from recent studies were relatively lower than reports from urban areas and were in the range of 8.2–25.3% [12, 15, 21, 22]. In general, the prevalence in our study was higher than most studies conducted in the country [8, 10, 23]. The proportion of hypertension increase with increasing age and the prevalence was also increasing with time compared with the previous study.

Studies conducted in different Sub-Saharan Africa (SSA) countries also showed different ranges of prevalence depending on the population studied. For example, in Nigeria prevalence of HTN was reported in the range of 25. 9%–35.4%; a report from Mozambique showed a prevalence of 33. 4% and similarly a report from Tanzania showed a 31% prevalence of HTN [5–7]. The urban-rural dichotomy in the prevalence of hypertension was also substantiated by a recently published cross sectional study in four SSA countries which showed an age adjusted prevalence of 23.2–25.8% in urban, 20.5% in peri-urban and 8.7% in rural residents [24]. These findings indicate that hypertension is increasing in the SSA countries particularly in urban residents.

The higher overall prevalence of hypertension in males identified in this study also agrees with global surveys and previous studies conducted in Ethiopia [3, 7, 11, 13, 20]. While this was the case for the whole study population, in participants above 65 years, the prevalence of hypertension was rather higher in females. This is consistent with a recent global study which showed that the mean systolic and diastolic BP and prevalence of hypertension is similar in males and females above the age of 50 years in most countries [3].

Isolated systolic hypertension was significantly more common in elderly while isolated diastolic hypertension, while not common in general, occurred more in young adults. This could plausibly be explained by the development of age related arteriosclerosis and arterial stiffening disproportionately elevating the systolic blood pressure in elderly.

Amongst those taking antihypertensive at the time of the study only about half had controlled blood pressure. Moreover, only 47% of the participants had ever had any kind of blood pressure measurement to the best of their recall. The remaining over half of the study participants didn’t remember for ever having their blood pressure measured regardless of the purpose. This finding could imply that the knowledge, attitude and practice towards hypertension and its treatment is very poor in the community and the situation could justify the fact that hypertension is a silent epidemic in Ethiopia. Similar findings have been observed in previous studies conducted in Ethiopia and other SSA countries [7, 11, 13, 20]. A hospital based study conducted five years a go to assess the adherence to antihypertensive therapy in University of Gondar Hospital found that only 64. 6% of patients were adherent to treatment and only 46. 6% had controlled BP [25]. Though this study didn’t assessed the prevalence of hypertension induced end organ damages, with such high occurrence of HTN, low rate of screening and poor control of HTN among those taking ant-hypertensive treatments, it is logical to expect an early onset of end organ damages in this community leading to increased cardiovascular morbidity and mortality.

In addition to male sex and older age, hypertension was strongly associated with being born and raised in urban areas. A number of other studies have also shown the urban-rural gradient in the prevalence of hypertension [12, 21, 24]. This may be explained by the environmental and life style factors associated with living in urban areas. Widowed and separated participants had also a higher prevalence of hypertension which may be the result of the psychosocial and economic stress that they have to bear with being a widow.

A history of daily alcohol consumption in the preceding one year was found to be independently associated with occurrence of hypertension in this study. This could be partly explained by the various lifestyle related psycho-social, economic and physical stress expected to accompany daily alcohol users.

Hypertension was strongly associated with obesity and overweight; previous studies have also shown that increasing BMI is associated with high blood pressure [11, 26, 27]. The finding that normal BMI was found to be associated with HTN compared to under-weight BMI could trigger an interesting topic regarding the potential advantages and disadvantages of being underweight in the dimension of NCDs. Moreover, being widowed or separated were also found to be independently associated with HTN when compared to being single. Though this finding could open up a whole new topic for discussion, one plausible explanation for this could be the potential socio-economic and lifestyle changes that might accompany being widowed or separated.

## Conclusion

Hypertension is a huge public health threat to the population of Gondar city. It is largely a concealed epidemic with low rate of awareness, screening and treatment. The momentum with which hypertension is growing in the public is not matched with an appropriate prevention, control and treatment strategy by the health system. Therefore, hypertension in particular and cardiovascular disease in general deserve due attention from policy makers. Strategies to raise the awareness of the public on the gravity of the situation and implementing accessible care and treatment packages is of an at most importance to decrease the up surging morbidity and mortality from cardiovascular disorders.

## Abbreviations

BMI: Body Mass Index
BP: Blood pressure
DBP: Diastolic blood pressure
HTN: Hypertension
IDA: International Diabetic Association
IDH: Isolated Diastolic Hypertension
ISH: Isolated Systolic Hypertension
NCDs: Non-communicable disease
SBP: Systolic Blood Pressure
SSA: Sub-Saharan Africa
WHO: World Health Organization
